# Tibiocalcaneal arthrodesis using the Ilizarov fixator in compromised hosts: an analysis of 19 patients

**DOI:** 10.1007/s00402-021-03751-0

**Published:** 2021-01-23

**Authors:** Charlotte Reinke, Sebastian Lotzien, Emre Yilmaz, Yannik Hanusrichter, Christopher Ull, Hinnerk Baecker, Thomas A. Schildhauer, Jan Geßmann

**Affiliations:** Department of Trauma Surgery and General Surgery, BG University Hospital Bergmannsheil, Ruhr University Bochum, Bürkle-de-la-Camp-Platz 1, 44789 Bochum, Germany

**Keywords:** Arthrodesis, External fixator, Ilizarov, Limb salvage, Tibiocalcaneal

## Abstract

**Introduction:**

Salvage of joint destruction of the tibiotalar and subtalar joint with necrosis or infection of the talus in compromised hosts is a challenging problem. In these cases, tibiocalcaneal arthrodesis using the Ilizarov external fixator represents a possible alternative to amputation. This retrospective study presents the results and complications of this salvage procedure.

**Materials and methods:**

Between 2005 and 2015, 19 patients were treated with tibiocalcaneal arthrodesis using the Ilizarov external fixator. Ten patients received tibiocalcaneal arthrodesis due to an acute or chronic infection with joint destruction. The other nine patients presented posttraumatic necrosis of the talus or Charcot arthropathy. In addition to demographic data, the time spent in the fixator, the major and minor complications and the endpoint of the consolidation were evaluated retrospectively. Furthermore, clinical outcomes were measured using the modified American Orthopedic Foot and Ankle Society (AOFAS) score.

**Results:**

The average time spent in the fixator was 22 (range 14–34) weeks. The average follow-up in 17 patients was 116 (range 4–542) weeks. Two patients were lost to follow-up. Complete osseous consolidation was achieved in 14 out of 19 patients. One patient presented partial consolidation, and in four patients, pseudarthrosis could be detected. The mean modified AOFAS score at the final follow-up was 53 out of 86 possible points.

**Conclusion:**

Tibiocalcaneal arthrodesis using the Ilizarov fixator is a possible salvage procedure even in compromised hosts. However, the healing rates are below the rates reported in the literature for tibiotalar arthrodesis in comparable clinical situations.

## Introduction

Since the introduction of compression arthrodesis by Charney in 1951, several different surgical procedures have been described as treatment options for ankle destruction of various etiologies [1]. Today, external Ilizarov arthrodesis represents an established and effective procedure in compromised hosts with acute or chronic infections, soft tissue defects, axial malpositions (varus/valgus) and comorbidities (diabetes mellitus, polyneuropathy, peripheral vascular disease, and alcohol abuse) [1–14]. Some authors even consider the Ilizarov method to be the “gold standard” in these difficult situations [2]. Tibiotalar arthrodesis has been described in most of these cases [2–11, 13, 14]. However, in patients with severe talus destruction due to infection or necrosis, talectomy might be required. In these patients, tibiocalcaneal arthrodesis is necessary to potentially achieve a painless stable limb [2, 3, 7, 8, 12–18]. However, only a few studies in the literature described tibiocalcaneal arthrodesis using the Ilizarov fixator. These were mostly small case series (2–12 patients) and focused mainly on tibiotalar arthrodesis (Table 1). Isolated studies on tibiocalcaneal arthrodesis using this procedure are very rare, and the number of complications is large [2, 3, 7, 8, 13, 15].

**Table 1:** Comparison of the results of this study with the existing literature on tibiocalcaneal arthrodesis

Authors	Patients	Number with infection	Time spent in fixator (months)	Consolidation	Consolidation in cases of infection	Mean AOFAS score (max 86)
Johnson et al. 1992 [3]	4	4	7	3	3	Not calculated
Zarutsky et al. 2005 [7]	2	1	9	2	1	Not calculated
Rochmann et al. 2008 [13]	11	11	7	9	9	65 (range 44–77)
El-Alfy et al. 2010 [8]	2	0	9	2	0	76 (range 78–74)
Khanfour et al. 2012 [2]	8	4	9	7	4	78 (range 70–85)
Kugan et al. 2013 [18]	12	?	?	8	?	Not calculated
This study	19	10	5	14	9	–
	7^a^	4	5	6	4	53 (range 25–68)^a^

^a^The AOFAS score was calculated from the seven patients in whom the score could be determined

Therefore, the purpose of the present study was to assess the outcomes of compromised patients who underwent tibiocalcaneal fusion using the Ilizarov technique. Does the Ilizarov fixator achieve good results for tibiocalcaneal arthrodesis? Are these results comparable to those described in the literature for tibiotalar arthrodesis?

## Methods

The present study was performed in accordance with the Declaration of Helsinki. Ethical permission for this study was obtained from the ethics committee of the University of Bochum (RUB), and informed consent was obtained from all patients before participation in the study (registration number 18-6582-BR).

All patients who underwent tibiocalcaneal fusion using the Ilizarov external fixator at our institution from January 2005 to December 2015 were retrospectively reviewed. To capture all patients with these criteria, a keyword analysis of all digitized files was performed by the author. The medical records of these patients were reviewed for the following factors: sex, age, associated relevant concomitant diseases, reason for arthrodesis, time spent in the fixator, complications and bony consolidation (Table 2). According to Katsenis, complications are considered minor when conservative therapy is sufficient and major when surgical revision is required [19]. The data were collected anonymously using Microsoft Excel^©^ Version 14.7.7. The exclusion criteria were as follows: (1) patients with isolated tibiotalar or subtalar arthrodesis and (2) patients with simultaneous tibiotalar and subtalar arthrodesis.

**Table 2:** Demographic data and outcomes of the 19 patients

Patient	Age	Pathology	Duration in frame (weeks)	Complications (major)	Revisions	Follow-up (weeks)	Achieved union	Modified AOFAS Score (max 86)	Comorbidity
1	59	Arthrosis	32	None	None	43	Yes x-ray	54	HTN, PNP
2	67	Charcot	19	None	None	7	Yes x-ray	–	Obesity
3	43	Charcot	16	None	None	44	No x-ray	–	Obesity, PNP
4	30	COM	18	None	None	8	Yes x-ray	–	COM, polytoxicomania
5	49	Acute infection	33	None	None	161	Yes CT	62	HTN
6	53	Charcot	14	None	None	0	Partial CT	–	DM, PNP
7	52	Acute infection	29	None	None	57	Yes CT	43	DM, PNP, alcohol abuse
8	60	Acute infection	19	None	None	25	Yes x-ray	62	Smoking, PVD
9	57	Arthrosis	14	None	None	68	No CT	25	Smoking, HTN
10	68	Arthrosis	22	None	None	39	Yes CT	–	HTN, DM, obesity
11	75	Acute infection	22	None	None	99	Yes x-ray	–	DM
12	61	COM	34	None	None	27	Yes x-ray	–	DM, HTN, PNP
13	59	Charcot	17	2	3	106	Yes CT	–	PNP, rheumatoid arthritis
14	74	Acute infection	27	None	None	341	Yes CT	58	HTN
15	62	Acute infection	20	None	None	4	Yes CT	–	DM, PNP, HTN
16	68	Arthrosis	19	None	None	32	No CT	–	PNP
17	61	Charcot	18	1	1	19	Yes CT	68	DM, PNP
18	75	COM	21	None	None	28	No CT	-	COM
19	60	Acute infection	17	None	None	0	Yes CT	–	–

The study included a total of 19 patients (10 men and 9 women), with an average age of 60 (range 30–75) years

*F* female, *M* male, *COM* chronic osteomyelitis, *DM* diabetes mellitus, *PNP* polyneuropathy, *PVD* peripheral vascular disease, *HTN* hypertension, *CT* computed tomography, *AOFAS* American Orthopedic Foot and Ankle Society

**Figs. 1:**
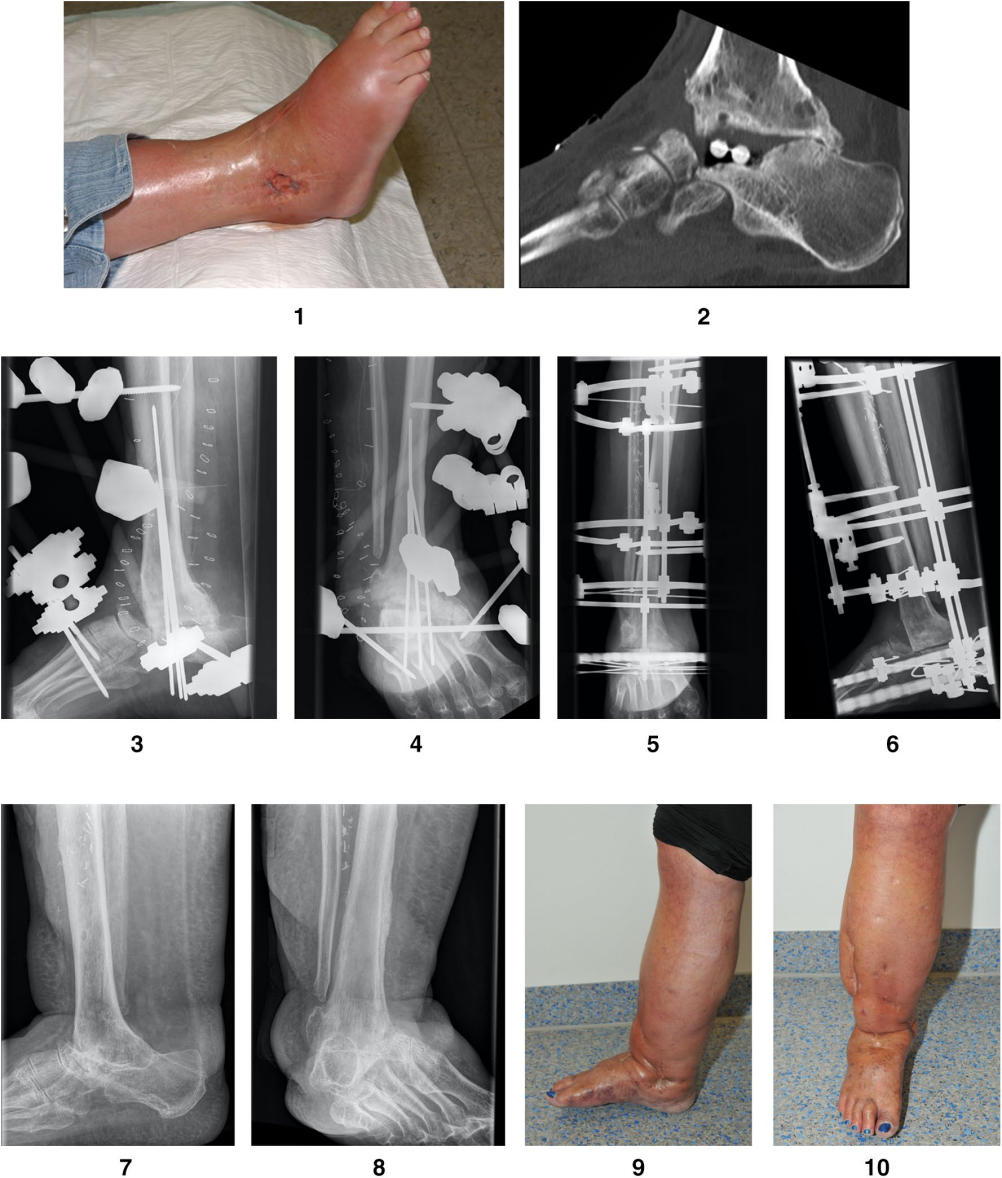
** Figs. 1, 2**: Patient 1—a 49-year-old woman presented with florid infection and destruction of the subtalar joint and talus and preexisting arthrodesis of the ankle after an initial fracture and multiple operations. When the patient was referred to our hospital, antibiotic chains were still present in the subtalar joint. **Figs. 3, 4**: Patient 1—x-ray image after talus resection, extensive debridement and placement of an AO fixator. The talus was completely destroyed due to infection, which is why it was completely removed. After initial VAC therapy, a plastic flap covering with the anterior lateral thigh (ALT) flap was necessary. **Figs. 5, 6**: Patient 1—after the soft tissue was successfully covered, the Ilizarov fixator was installed. **Figs. 7, 8**: Patient 1—after the fixator had been worn for 33 weeks, it was removed and consolidation was observed. **Figs. 9, 10**: Patient 1—clinical picture at the last follow-up examination after 121 months. The woman was able to walk, and the AOFAS score was 62 of 86 possible points

**Figs. 2:**
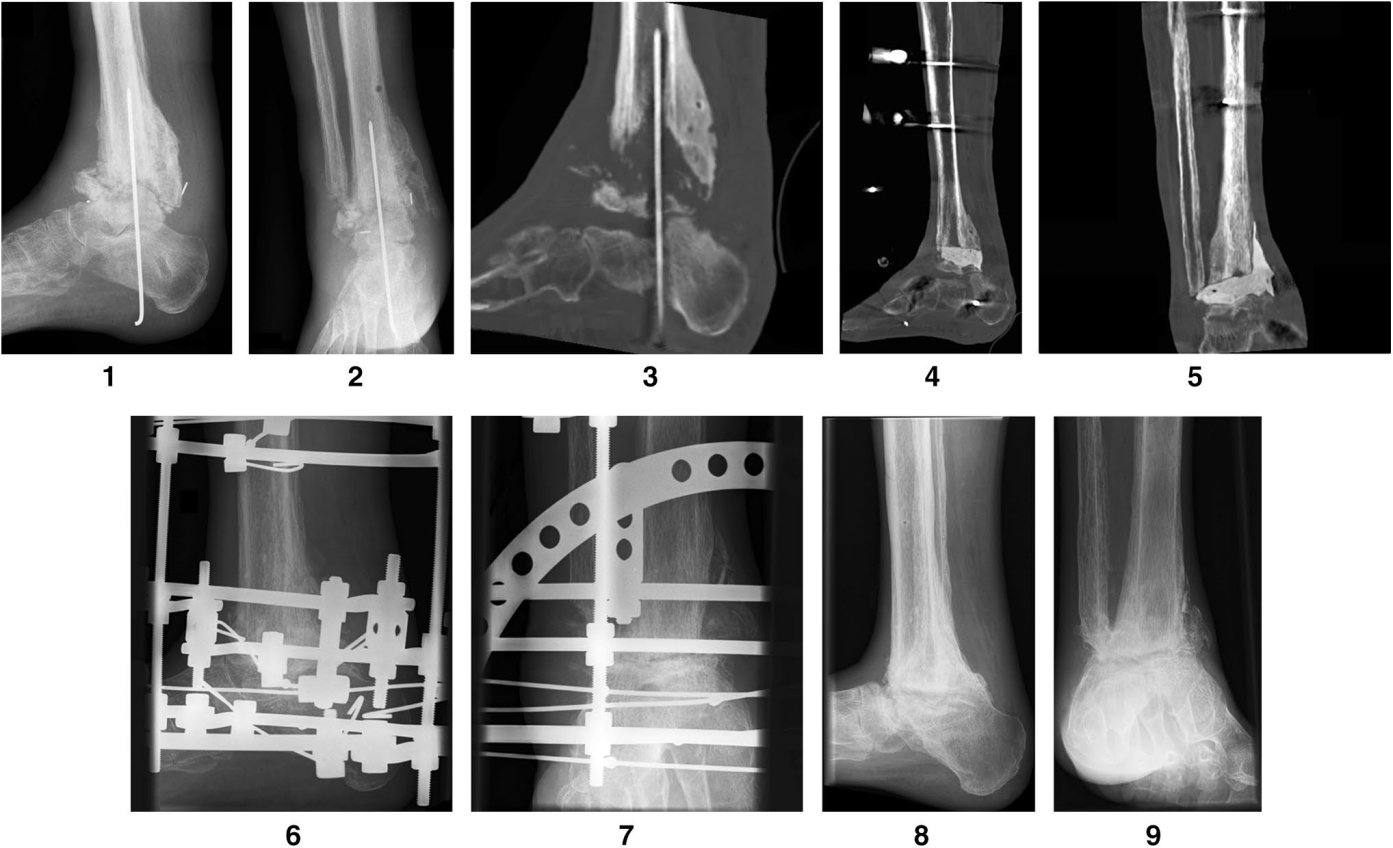
**Figs. 1, 2, 3**: Patient 2—a 61-year-old patient with previous Weber C fracture and chronic osteomyelitis and multiple previous operations abroad presented with acute soft tissue and bone infection. The patient had known diabetes mellitus, polyneuropathy and coronary artery disease. **Figs. 4, 5**: Patient 2—in the first procedure at our facility, resection of the talus and detailed debridement were carried out while leaving the talus head. A two-stage procedure with insertion of a cement spacer containing antibiotics and attachment of an AO fixator was performed. **Figs. 6, 7**: Patient 2—after addressing the infection and conditioning the soft tissue, the Ilizarov ring fixator was installed. **Figs. 8, 9**: Patient 2—with bony consolidation, the Ilizarov fixator was removed after 7 months

The study included a total of 19 patients (10 men and 9 women), with an average age of 60 (range 30–75) years. Using the Cierny/Mader staging system, three patients were classified as having type IV osteomyelitis [20], and seven patients suffered from a florid, fulminant infection with joint destruction. The reasons for this were posttraumatic soft tissue defects in diabetic foot syndrome and periprosthetic infection in an ankle prosthesis. Of the remaining patients, four presented with posttraumatic arthrosis/necrosis and five suffered from Charcot arthropathy with talar necrosis. All patients also showed poor soft tissue condition, with pronounced scar tissue, fistula formation, soft tissue abscesses and/or necrosis of the skin (patient 1, Figs. 1, 2). Concomitant diseases were found in all patients, of whom 13 suffered from relevant diseases such as diabetes mellitus, polyneuropathy, polytoxicomania and/or peripheral arterial occlusive disease (patient 2, Figs. 1, 2, 3). Another risk factor was nicotine abuse in three patients (Table 2). As part of the operative treatment, all patients underwent resection arthroplasty of the distal tibia, including resection of the lateral and medial malleolus through a medial and lateral approach. The resection height in the area of the distal tibia is determined according to the proportion of the destroyed bone. The distal tibia is resected until non-sclerotic and well-perfused bone is visible.

Then, the talus and cartilage of the calcaneus and navicular bone were removed, and extensive debridement was performed. To achieve simultaneous tibionavicular arthrodesis over the leading edge of the tibia, the anterior prominence of the tibial plafond was also decorticated. In all cases except two, the whole talus was removed. In these two patients, the talus head and thus the talonavicular joint could be preserved (patient 2, Figs. 4, 5). Bone grafts were used in four patients with posttraumatic arthrosis/necrosis and in three patients with Charcot arthropathy. The graft was always taken from the resected fibula/tibia and the iliac crest. In two cases involving soft tissue defects, an AO fixator was used first, and a temporary wound vacuum-assisted closure (VAC) was installed (patient 1, Figs. 3, 4). In two other cases involving soft tissue defects, both a temporary wound VAC and the Ilizarov fixator were attached simultaneously. Three of the tissue defects were successfully treated by wound VAC with subsequent mesh graft coverage. In the fourth patient, a plastic flap covering with an anterior lateral thigh (ALT) flap was necessary. After successful soft tissue conditioning, the AO fixator was removed, and the Ilizarov frame was attached (patient 1, Figs. 5, 6, patient 2, Figs. 6, 7). In three patients with acute infection, a two-stage procedure with extensive debridement and insertion of a cement spacer containing calculated standard antibiotics (2 g vancomycin and 0.55 g gentamycin per 40 g cement) and attachment of an AO fixator was performed. After 30 days, the cement spacer was removed, and the Ilizarov fixator was placed for arthrodesis. In all other cases, fusion was performed immediately as a single-stage procedure. The Ilizarov frame consisted of three to four rings that were fixed by means of four half pins and two olive wires in the tibia, two olive wires in the calcaneus and wires for fixing the metatarsus. It was preassembled preoperatively and applied en bloc. All septic patients initially received either a calculated antibiotic treatment or antibiotics in accordance with the resistogram for a total of six weeks. All patients were allowed a full axial load, avoiding the rollover process to prevent the metatarsal wire from breaking, which is important for overall stability of the frame and in the arthrodesis zone. To enable the rollover process, in six patients, a rolling sole was installed under the fixator. To avoid the development of claw toes, the patients received toe reins, which could be attached to the fixator in rest periods and overnight via rubber straps. The Hba1c was determined in three cases of seven patients with diabetes before the start of treatment and was too high in two cases with 7.1% and 7.5% (Ref. range 4.0–6.0%). The third patient was within the target range with 5.2%. Daily blood sugar profiles were determined for the other patients. The sugar was poorly controlled in two patients before the start of treatment. The patients with poorly controlled diabetes received a diabetes control. After discharge, biweekly tensioning of the fixator for compression arthrodesis took place during our consultation (the fixator was compressed by 2 mm each time). Radiological control was performed every four weeks.

After removal of the ring fixator, the average clinical/radiographic follow-up period was 116 (range 4–542) weeks in a total of 17 patients. Two patients were lost to follow-up. Bony union was confirmed clinically and radiographically. Clinical signs included no motion at the fusion site. Union was defined radiographically using plane radiographs in four projections or computed tomography (CT) scans. In 12 cases, a CT was performed to verify the consolidation. Of the remaining seven cases, there was a clear consolidation in the X-ray in six cases and a clear nonunion in one case. In the case of nonunion, the patient did not want any further surgical treatment, which is why the CT was not used.

The outcome at final follow-up was assessed using the modified American Orthopedic Foot and Ankle Society (AOFAS) Score [21]. It was modified because of the elimination of tibiotalar and subtalar motion. Therefore, the maximum possible score for our patients with a successful tibiocalcaneal fusion was 86 [9, 12].

## Results

The average time in frame was 22 (range 14–34) weeks. Bony arthrodesis was primarily achieved in 14 out of 19 patients (patient 1, Figs. 7, 8, patient 2, Figs. 8, 9). One patient presented partial consolidation (only 40–50% consolidation in the sagittal and coronal CT slices), and in four patients, pseudarthrosis could be detected. In these patients, a conservative procedure was carried out by using carbon orthotic adaptation. During treatment in the fixator, local pin infections occurred in all patients, but all of them could be treated locally by pin care and, if necessary, stab incision and oral antibiotics. The metatarsal pin broke in five patients, and two of these pins could be refixed. The other patients were treated by pin removal. No further treatment was necessary in these cases because of advanced consolidation. Two of the patients had a pin break despite the attached rolling sole.

Three major complications occurred in two patients. In patient number 13 (Table 2), after 7 weeks of fixator time, a bony outbreak of a half pin in the area of the tibial shaft occurred. The broken pin was removed, and two new half pins were inserted. Six days later, six olive wires had to be changed because of an unstable fixator. After removal of the fixator (after 17 weeks), there was bony consolidation in the area of arthrodesis, but the patient complained about pain in the tibial shaft area where the outbreak occurred. Therefore, surgical treatment was carried out by means of plate osteosynthesis. After 6 weeks, consolidation could be established.

In patient number 17, 3 months prior to fixator removal, a forefoot pin breakage occurred. This could not be fixed, so a surgical change was necessary due to an unstable situation. After 18 weeks of fixator time, bony consolidation could be achieved.

Patients who suffered from infectious destruction showed no signs of recurrent infections during the follow-up period of 116 (range 4–542) weeks on average (patient 1, Figs. 9, 10).

The AOFAS score was fully determined during the final follow-up for only 7 out of 19 patients. The mean modified score was 53.

Two patients out of seven reported no limitation in their activities of daily living, and three patients were able to walk more than six blocks. Severe limitations of daily and recreational activities were reported by two patients. Of seven patients, four patients reported having mild occasional pain, two reported moderate daily pain and one patient reported no pain. No patient complained of severe pain.

## Discussion

In a study from 1992, Johnson et al. were the first to perform arthrodesis in a small case series (six patients) using the Ilizarov fixator [3]. Arthrodesis was achieved in three out of four tibiocalcaneal arthrodesis and two tibiotalar arthrodesis procedures.

Since then, there have been many studies of ankle arthrodesis with the Ilizarov fixator, and this procedure is now considered an established standard procedure in complicated patients, such as patients with chronic osteomyelitis, acute infection, diabetes mellitus and/or compromised soft tissue. Consolidation rates are reported to be between 75 and 100% [2–6, 8–11, 13, 14]. However, most studies analyzed patients who underwent tibiotalar arthrodesis. Salem et al., Yanuka et al., Gessmann et al. and Fragomen et al. reported consolidation rates of 95%, 100%, 86.5% and 83%, respectively, exclusively via tibiotalar stiffening [5, 6, 9, 10]. In the event of destruction of the tibiotalar and subtalar joint in cases involving a vital talus, there is also the possibility of simultaneous arthrodesis of both joints using an Ilizarov fixator [22] and in the case of Charcot arthropathy, Wirth et al. described the possibility of foot reconstruction with limb preservation via Ilizarov fixator [23].

However, tibiotalar arthrodesis or simultaneous arthrodesis is not possible in cases involving posttraumatic talar necrosis or in cases involving talar destruction with infection or Charcot arthropathy. Nevertheless, to guarantee preservation of the extremities, even in complicated patients, a talectomy and tibiocalcaneal arthrodesis must be performed. Johnson et al. reported on four tibiocalcaneal arthrodesis procedures in a total of six patients in 1992, and successful fusion could be achieved in three out of four cases [3]. In the following years, other authors also reported on tibiocalcaneal arthrodesis, but each study included mainly patients with tibiotalar arthrodesis via the Ilizarov fixator. For example, Zarutsky et al. reported only two cases of tibiocalcaneal arthrodesis in his study consisting of 43 cases. In both cases, consolidation was described in aseptic patients [7]. Additionally, El-Alfy et al. reported on only two tibiocalcaneal arthrodesis procedures in a total of 12 patients with avascular talar necrosis. Both cases achieved bony fusion [8]. Khanfour et al. and Kugan et al. reported tibiocalcaneal arthrodesis in only eight out of 30 and 12 out of 48 patients, respectively. The consolidation rates were 87% and 67%. However, Khanfour et al. did not report on patients with an acute infection [2, 13]. One study by Rochmann et al. from 2008 treated isolated tibiocalcaneal stiffening via an Ilizarov fixator. In a septic patient population of 11 patients, successful fusion was described in nine cases [12]. In our mixed septic/aseptic study, the rate of successful bony fusion was similar, with 14 out of 19 patients.

Our study is in line with the existing literature (i.e., Rochmann et al.) reporting worse results than studies analyzing patients with tibiotalar arthrodesis [5, 6, 9, 10, 12]. Kugan et al. also described a higher failure rate after loss of the talus in his study [13]. The calculated modified AOFAS score (max 86 points) was 53 points on average (min 25; max 68) in seven patients in this study, which is below the reported score of 65 points (eleven patients) in the study by Rochmann et al. [12]. Khanfour et al. also reported a higher score (78) in eight patients. Further studies are needed to determine whether, for example, concomitant leg extension has an impact on this score, which was done in eight patients in the Rochmann et al.’s study and in seven patients in the Khanfour et al.’s study [2, 12]. However, simultaneous leg length compensation extends the fixator’s wearing time, increasing the risk of potential complications [2, 5, 10, 12]. The average time spent in the fixator was seven months in the Rochmann et al.’s study and nine months in the Khanfour et al.’s study [2, 12]. Compared with an average of five months in our study, a significantly longer wearing time must be discussed with the patient. The number of major complications was three out of 19 patients in our study. This is in accordance with the results of tibiotalar arthrodesis reported by Gessmann et al. (16% in 37 patients) and is below the rate reported by Zarutsky et al. (51.2% in 43 patients) [7, 9]. It is also below the rates from studies of tibiotalar arthrodesis with concomitant lengthening of the limb (Fragomen et al. 32%; Salem et al. 36%) [5, 10]. In the Rochmann et al.’s study, there were also three major complications in 11 patients, all of which were due to concomitant lengthening. Simultaneous leg lengthening was not performed in our study due to the comorbidities in our patient population and the associated increased risk of complications such as insufficiency or delayed maturation of regenerated bone in proximal tibial transport [12, 24]. In our study, the focus was on limb preservation, and a difference in leg length was tolerated. The difference in leg length could be compensated well in all patients with orthopedic footwear.

Comorbidities and age could also be further reasons for the comparatively poorer modified AOFAS scores in our study. The patients in our study were on average 60 years old when the fixator was applied, compared to 44 years for Rochmann et al. and 40 years for Khanfour et al. and all had relevant comorbidities such as diabetes, obesity or nicotine use [2, 12]. In six cases, there were also soft tissue defects such as fistula formation, soft tissue abscesses and/or necrosis of the skin, making installation of a temporary wound VAC, mesh graft coverage and a plastic flap covering necessary. Khanfour et al. excluded patients with uncontrollable diabetes, a lack of plantar sensitivity, and/or peripheral vascular disease, and the study by Rochmann et al. did not provide details on soft tissue or comorbidities [2, 12].

This study has several limitations. The study sample of 19 patients was small, and the study had a retrospective design. Therefore, therapeutic recommendations should be made carefully based on our results. Postoperative CT scan was not performed as standard in all patients. Furthermore, two patients were lost to follow-up, and the AOFAS score was completed in only seven out of 19 patients, because it was not determined by default for all patients as part of the follow-up. Furthermore, the use of the AOFAS score is currently no longer recommended [25].

We treated patients from all over the country, which might be a reason why the patients were not available for follow-up.

There was also no long-term clinical follow-up for the evaluation of subjective patient satisfaction.

However, the results presented here show that this method represents an alternative to amputation for selected patients. However, decisions will require a detailed explanation of the long and complex treatment as well as the procedural complications on a case-by-case basis.

## Conclusion

Tibiocalcaneal arthrodesis using an Ilizarov external fixator can be a possible salvage procedure even in compromised hosts. However, the fusion rates are below the rates reported in the literature for tibiotalar arthrodesis in comparable clinical situations.

## Abbreviations

AOFAS: American Orthopedic Foot and Ankle Society
VAC: Temporary wound vacuum-assisted closure
ALT: Anterior lateral thigh flap
CT: Computed tomography
